# Exploring vasculogenesis in the normal human kidney and clear cell renal cell carcinoma: insights from development to tumor progression and biomarkers for therapy response

**DOI:** 10.3389/fonc.2024.1375190

**Published:** 2024-04-30

**Authors:** Andrei Alexandru Cosma, Mihaela Pasca Fenesan, Alexandru Nesiu, Eugen Melnic, Adela Maria Ferician, Ovidiu Catalin Ferician, Emil Ceban, Simona Sarb, Anca Maria Cimpean

**Affiliations:** ^1^ Department of Microscopic Morphology/Histology, Victor Babes University of Medicine and Pharmacy, Timisoara, Romania; ^2^ OncoHelp Hospital, Timisoara, Romania; ^3^ Doctoral School in Medicine, Victor Babes University of Medicine and Pharmacy, Timisoara, Romania; ^4^ Department Medicine, Discipline of Urology, Vasile Goldiş Western University, Arad, Romania; ^5^ Department of Pathology, “Nicolae Testemitanu” State University of Medicine and Pharmacy, Chişinău, Moldova; ^6^ Medlife Medicis Clinics, Timisoara, Romania; ^7^ Department of Orthopedy and Traumatology/Urology, Victor Babes University of Medicine and Pharmacy, Timisoara, Romania; ^8^ Department of Urology and Surgical Nephrology, Nicolae Testemitanu State University of Medicine and Pharmacy, Chisinau, Moldova; ^9^ Laboratory of Andrology, Functional Urology and Sexual Medicine, Nicolae Testemitanu State University of Medicine and Pharmacy, Chisinau, Moldova; ^10^ Center of Expertise for Rare Vascular Disease in Children, Emergency Hospital for Children Louis Turcanu, Timisoara, Romania

**Keywords:** vasculogenesis, endothelial progenitor cells, clear cell renal cell carcinoma (cc RCC), metastases, tumor microenvironment, blood vessels

## Abstract

Vasculogenesis, which refers to the development of blood vessels from precursor cells, is a process that occurs predominantly during early embryonic life. It plays a crucial role in the establishment of the primitive vascular network. Vasculogenesis diminishes throughout the fetal vascular remodeling process, giving way to angiogenesis, which becomes the predominant mechanism after birth. At first, the development of the kidney’s blood vessels depends on vasculogenesis, and then both vasculogenesis and angiogenesis happen simultaneously. Both processes are necessary for the normal development of the renal vasculature. Although the kidneys are highly vascularized, our understanding of normal kidney vasculogenesis is still incomplete. This lack of knowledge may explain the limited data available on the role of vasculogenesis in the progression and spread of renal cancers. In other types of cancer, researchers have well documented the phenomenon of tumor vasculogenesis. However, there is currently limited and fragmented information about the occurrence of clear-cell renal cell carcinomas (cc-RCC). In this article, we provide a comprehensive review of the current understanding of normal kidney vasculogenesis and vasculogenic pathways in clear cell renal cell carcinoma (cc-RCC). We specifically focus on cellular precursors, growth factors, and the influence of the normal and tumor environments on these processes. It will carefully look at how tumor vasculogenesis might affect the growth and metastasis of clear cell renal cell carcinoma (cc-RCC), as well as how it might affect the effectiveness of drugs and the development of therapy resistance.

## 1 Normal vasculogenesis

Vasculogenesis and angiogenesis (1) are the two processes that control the growth of blood vessels in embryos so that the primitive vascular plexus can form. Even though they both lead to the formation of blood vessels, vasculogenesis and angiogenesis are two separate and well-defined processes by which the body creates and repairs its vascular bed in healthy and sick situations (2–4). Noden (5), Poole, and Coffin (6–8) paved the way for subsequent seminal works by Risau and *colab*. in the field of vasculogenesis (9–13). represented the milestones in the description and accurate understanding of normal vasculogenesis in early embryonic life. Several authors (14, 15) have described how vasculogenesis depends highly on the peculiarities of each organ microenvironment, making it an organ-specific process.

### 1.1 Overview on embryonic and adult normal vasculogenesis

Vasculogenesis (16) defines that the first development of embryonic blood vessels happens on its own in the mesenchyme of the yolk sac wall, the chorion, and the embryonic disc. Researchers intensely study normal vasculogenesis using several different experimental models, ranging from zebrafish (17) or chick embryo chorioallantoic membrane (18–20) to organoids (21) or droplet-based scRNA-seq profiling (22). Embryonic vasculogenesis consists of two steps: an extraembryonic step, which occurs in the primary yolk sac, and an intraembryonic step (23).

#### 1.1.1 Extraembryonic vasculogenesis

The human primary yolk sac mesoderm is the first extraembryonic site of endothelial precursor differentiation, starting 2.5 weeks post-conception (WPC) (24) followed by the extraembryonary primitive capillary plexus formation by vasculogenesis. Two sources of endothelial cells were identified during primary yolk sac extraembryonic vasculogenesis: yolk sac mesodermal cells (non-hematopoietic origin) and erythro-myeloid progenitors’ cells (with hematopoietic origin) (23, 24). The commitment of mesodermal cells through the endothelial colony-forming cells (ECFCs) lineage is part of early endothelial cell differentiation from mesodermal cells (25). A study by Bruveris et al. (25) used a human model of yolk sac hematopoiesis to look at how human blood and endothelial lineages separate early on. Their model mimicked yolk sac blood islands, allowing them to study the divergent development of both endothelial and hematopoietic precursors that start from mesodermal cells. The Vascular Endothelial Growth Factor (VEGF) and Fibroblast Growth Factor 2 (FGF2) govern mesodermal cell commitment through the ECFC lineage, with SOX17 expression initiating during their differentiation into the endothelial cell phenotype. They defined an intermediate stage of SOX17-CD34+CD43-blast colony (hemangioblast stage) able to give rise to both angioblasts having SOX17+CD34+CD43-phenotype and hematopoietic precursors with SOX17-CD34+CD43+phenotype (25). SOX17+CD34+CD43- angioblasts upregulate a series of genes (including APLNR, CDH5, FLT1, ESAM, EFNB2, and CD93) and transcription factors (including SOX7, SOX17, SOX18, ERG, ETS1, ETS2, and HOPX) in their gene expression profile (26). Some genes and transcription factors upregulate during malignant progression. Endoglin (CD105) and Flk1 upregulate and co-localize in the yolk sac angioblasts responsible for primitive vessel formation in the next step of extra-embryonic vasculogenesis (27). Yolk sac angioblasts (which are negative for VE-cadherin but positive for CD133, CD34, and VEGFR2) can divide and move around easily (28). They keep changing into endothelial progenitor cells (EPCs, VE-cadherin positive, Ki67 positive), and then they fully change into mature endothelial cells that are not doing anything (28). Endothelial progenitor cells lose their ability to divide (they become Ki67-negative) as they mature and form endothelial junctions by acquiring PECAM1 (28). Endothelial junctions’ development, together with a decrease in proliferative activity, induces the aggregation of derived mature endothelial cells into cords. A cord is a string of angioblasts and EPCs that lack a vessel lumen. Tip cells maintain proliferative and migratory states, while stalk endothelial cells are responsible for lumen formation. Through the growth of pseudopodia, tip cells keep the migratory phenotype and continue to direct angiogenic growth factors. Stalk cells have a high proliferative phenotype, and they are the main source of endothelial cells, contributing not only to the length of future blood vessels but also to lumen formation. The mechanisms for lumen development are complex and still controversial. Researchers strongly debate several models of vascular cord tubulogenesis (lumenization) (29–32). The interplay between tip and stalk cells (32) highly governs vasculogenic tubulogenesis. In response to rapid growth-related hypoxia, the embryonic mesenchyme releases a lot of angiogenic growth factors. These factors keep tip cells from the yolk sac primitive vascular cords moving through the embryonic disc. Vasculogenic tubulogenesis includes two mechanisms: EC hollowing and cord hollowing (29). Aligned ECs form the lumen through intracellular vesicles fusing. Both tip and stalk cells have the ability to create intracytoplasmic vacuoles, which will subsequently fuse and initiate lumen formation (30). It is thought that cord hollowing happens when ECs and their intercellular junctions move around, causing slits to form and connect between ECs (29). Tip endothelial cells can change the extracellular matrix (ECM) composition and facilitate tunnel-like structures to guide the formation of new vascular cords (29). Certain extracellular matrix (ECM) components strongly stimulate the process of vascular tube morphogenesis, while others have inhibitory effects. It is important to note that collagen type I and fibrin are common parts of the extracellular matrix (ECM) in adult animals. They play a significant role in shaping the shape of blood vessels in three-dimensional environments (29).

#### 1.1.2 Intraembryonic vasculogenesis

**Figure 1:**
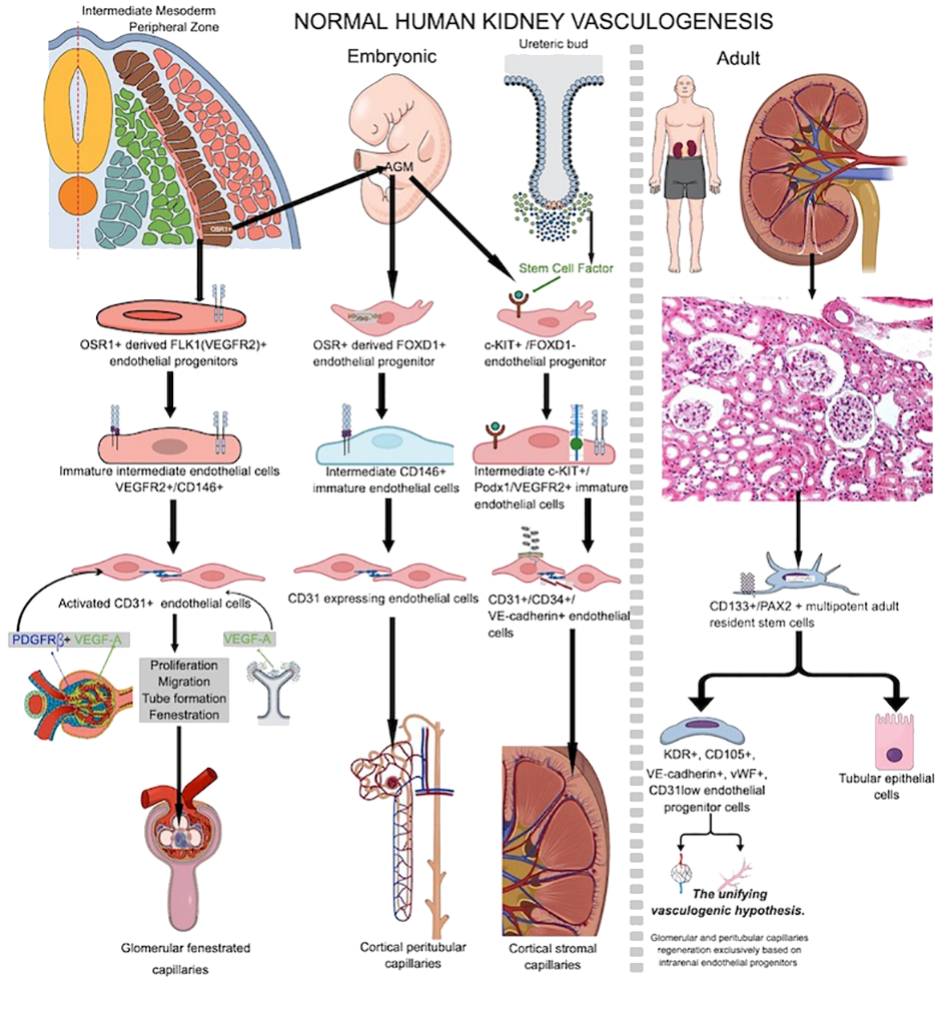
Schematic overview of normal human embryo and adult vasculogenesis inside renal tissue during prenatal kidney development or vascular injury repair in adult life. Note the heterogeneous source of endothelial progenitor cells participating into kidney embryo vasculogenesis compared to endothelial sources paucity in adult kidney which is most probably restricted to resident stem cells inside adult kidney stroma.

In 2008, Wang et al. described a mesenchymal stem cell population in the human yolk sac and human embryo in the early stages of its development (33). There are mesenchymal stem cells (MSCs) that are not hematopoietic or endothelial, but they are fibroblastoid. These cells can move inside the embryo and populate the aorta-gonad-mesonephros region (AGM), where they make up about 0.3% of all the cells in that region (33). The same authors said that these MSCs found in the mesenchyme around the human dorsal aorta can pick up CD31 and CD34 endothelial markers and can create capillary plexuses (33). This is one of the few pieces of evidence of human intraembryonic vasculogenesis. Before, Marshall et al. found a layer of CD34-positive cells on the ventral side of the human dorsal aorta that had high levels of tenascin-C and fibronectin (34). The authors suggested that CD34 stromal positive cells may generate a common precursor for both endothelial and hematopoietic lineages, but they mainly focused on hematopoiesis rather than intraembryonic vasculogenesis in their study (35). Most studies on this topic have been conducted on animal experimental models (36, 37) rather than human embryonic tissues, leading some authors to consider the ventral side of the dorsal aorta as the first site of intraembryonic vasculogenesis due to the old concept that yolk sac endothelial precursors invade embryonic discs. Researchers now widely acknowledge that the vasculogenesis process varies significantly among species (37). A new idea about how blood vessels form in human embryos says that it starts at 3 to 4 PCWs and spreads to several mesenchymal sites inside the embryo, but the process is different depending on the organ (**Figure 1**). The most active site of intraembryonic endothelial progenitors’ differentiation is the aorta-gonad-mesonephros region on the ventral wall of the human dorsal aorta (24) followed by the mesenchyme of vital organs like the heart (38, 39), liver (40, 41), lungs (42), nervous system (43) or kidney tissue (44, 45). In addition to the “classic” concept of vasculogenesis (based on *de novo* blood vessel formation starting from angioblast-derived endothelial cells of mesodermal origin), several papers described a particular type of vasculogenesis named hemo-vasculogenesis (46) where *de novo* blood vessel development starts from endothelial cell precursors differentiated from hematopoietic cells or erythron-myeloid precursors (EMPs) (23). Differentiated endothelial cells from EMPs can organize into cords, help make lumens, and insert between existing blood vessel endothelial cells to heal after endothelial injury in adults. Endothelial colony-forming cells (ECFCs) are a subset of endothelial progenitor cells that exhibit clonogenicity progenitor characteristics and are found in cord blood, but researchers have also isolated them from various organs such as the lungs, liver, heart, or kidney (47–52). Because they are present in several organs in both adult and embryonic life, ECFCs are considered tissue-vascular resident cells with progenitor potential. Their presence and description in the human adult organ stroma strongly support postnatal local vasculogenesis. Some indirect evidence from the literature suggests that endothelial precursor heterogeneity may be responsible for several vasculogenesis mechanisms that depend on the type of endothelial precursor that is used.

### 1.2 *De novo* blood vessels development in human embryo and adult kidney

#### 1.2.1 Prenatal renal vasculogenesis

During kidney organogenesis, the intermediate mesoderm (IM) differentiates to make the pronephros, mesonephros, and metanephros. These are three separate organs for getting rid of waste. The metanephric kidney persists postnatally and undergoes further development to establish a fully functional kidney in adulthood. Metanephros differentiation occurs when the metanephric mesenchyme (MM) and the ureteric bud (UB) lineage interact. Posterior intermediate mesoderm (PIM) generates the MM, while a more anterior intermediate mesoderm (IM) gives rise to the UB lineage, comprising the collecting system (53, 54). The metanephric kidney’s development is dependent on complex signaling pathways and cell-to-cell contact between at least four main types of progenitor cells in the nephrogenic zone (55). These cell types come from the ureteric bud, the nephron, the stromal, and the endothelial progenitors. The renal vasculature’s primary source is the development of endothelial progenitors (angioblasts) into *de novo* capillaries (56). There exist distinct populations of both intra-renal and extra-renal endothelial progenitor cells (EPCs) (57). Researchers have described at least three different EPCs that contribute to renal vasculogenesis (56). Type 1 renal EPCs, which are a group of cells that express FLK1 (VEGFR2) (58), are found on the edge of the intermediate mesoderm, close to the stalk of the ureteric bud (UB). They originate from odd skipped related 1 (OSR1) multipotent progenitor cells from intermediate mesoderm (a common precursor for both glomerular epithelial cells and endothelial cells), together with other epithelial cells, which will contribute to nephrogenesis (57). As they develop through the endothelial lineage, they go through an immature stage marked by the expression of the melanoma cell adhesion molecule (MCAM+, CD146+) antigen. Platelet/endothelial cell adhesion molecule-1 (PECAM+, CD31+) (59, 60) governs their full maturation. These cells will migrate into the S-shaped body’s vascular cleft and then differentiate, forming the fenestrated glomerular endothelium in between 7 and 9 PCW (61). Due to low oxygen levels inside the kidneys, VEGF A is released by developing glomerulus nephrogenic progenitor epithelial cells and then by differentiated podocytes and mesangial cells. PDGFRβ, which is made by mesangial cells, helps the cells move. FOXD1 cells derive from intermediate mesoderm OSR1-positive cells and migrate from the AGM region into metanephric tissue, serving as the progenitor for cortical stromal cells (57). FOXD1+ renal cortical stromal cells also transition to MCAM+ and differentiate into endothelial cells through CD31 acquisition. These cells are then integrated into the peritubular capillaries, influencing the spatial arrangement of renal vasculature (62–64), and are regarded as the second type of renal EPCs. A third renal EPC subpopulation expressing c-kit resides in the renal cortical stromal compartment during the early stages of metanephric mesenchyme development, where they are chemoattracted by the stem cell factor secreted by ureteric bud cells (65–67). Renal EPC heterogeneity supports the phenotypic and metabolic diversity of adult renal endothelial cells recently reported by Dumas et al. (68), as well as their functional, therapeutic, and damage response heterogeneity (69). Vasculogenesis and hemovasculogenesis develop capillary networks in the developing kidney. VEGFA (secreted by NPCs and ureteric bud cells) and stem cell factor secreted by ureteric bud cells (55, 70) initiate renal EPC activation. When VEGF A binds to VEGFR2 and SCF binds to c-kit, it activates renal EPCs, which causes them to grow, move, and form tubes. Podocytes secrete VEGF A and class 3 semaphorin, recruiting ECs into the S shape of the glomerular development stage, where the glomerular capillary network begins to develop and interconnect with podocytes (58, 71). Renal stromal cells (64, 72) highly influence glomerular vasculogenesis. By expressing SFRP1, stromal cells support EC proliferation, migration, and tube formation (73). A platelet-derived growth factor beta (PDGF-β) from endothelial cells binds to a platelet-derived growth factor receptor beta (PDGFRβ) on stromal cells. Endothelial cells draw to the glomerulus and initiate capillary loop formation. In contrast, PBX1 in stromal cells manages when and where PDGFRβ is expressed in the cortex of the kidney. This helps keep the vascular system of the kidney stable (74, 75). Also, macrophages inside the nephrogenic zone interact with nascent vessels, facilitating vascular anastomoses. Therefore, they significantly impact the establishment of a functional network of blood vessels (76).

#### 1.2.2 Postnatal normal human kidney vasculogenesis

Postnatal normal human kidney vasculogenesis is less studied for the kidney. In 2005, Bussolati et al. (77) isolated CD133+/PAX2+ multipotent adult resident stem cells from adult human kidneys with a dual ability to differentiate in both nephrogenic epithelial cells and endothelial cells. *In vitro*, they differentiate through an endothelial lineage and get KDR, CD105, VE-cadherin, and vWF. However, they only get low amounts of CD31, and human HLA-1 class I antigen (77). Matrigel implantation of endothelial differentiated cells caused cords and tubes to become capillary-like structures (77). When implanted subcutaneously in SCID mice, these *in vitro* differentiated cells spontaneously organized *in vivo* functional vessels that interconnected with host vessels and became perfused. All the above evidence strongly supports the presence of adult human kidney-resident endothelial progenitor cells. Lake et al. conducted a recent study that comprehensively analyzed a diverse range of healthy reference kidneys and diseased kidneys. This analysis utilized multiple single-cell and single-nucleus assays, encompassing over 400,000 nuclei or cells. Spatial imaging technologies played a crucial role in the establishment of the primitive vascular network. The outcome of this study was the creation of a detailed cellular atlas consisting of 51 primary cell types, including both rare and previously unidentified populations (78). The authors of this atlas said that CD133+/PAX2+ progenitors were only found in the proximal and distal tubules of the adult human kidney. This supports what Bussolati et al. (77) had found earlier. Endothelial progenitor cells drive the development of avascular glomeruli in kidney organoids in experimental models, but the addition of these cells prompts the development of glomerular capillaries and the recruitment of host vessels by the organoids (79). Similar factors drive postnatal renal vasculogenesis as embryonic vasculogenesis (80). As of now, there is only direct evidence that vascular endothelial progenitor cells are present in the normal human adult kidney. There is no other direct evidence that the normal human kidney has a process of continuous or intermittent vasculogenesis. It seems that human adult kidney vasculogenesis is activated by repetitive hypoxic conditions during several pathologic conditions, and “the unifying vasculogenic hypothesis” of Fine et al. sustains that function restoration of surviving nephrons could be achieved by regeneration of the renal microvasculature alone (81–83) based on the intrarenal presence of a heterogeneous population of endothelial precursors (84, 85). Eighty-five percent of the glomerular and 69% of the peritubular endothelium sustained damage in a rat model of renal thrombotic microangiopathy induced by graft perfusion with antiglomerular endothelial cell (GEN) antibody. Four weeks after injury, intrarenal endothelial progenitors were primarily responsible for renal endothelial healing and the recovery of kidney function through inducing new capillary development, with less contribution from extrarenal endothelial cells (85). **Figure 1** shows a comparative presentation of embryonic and adult kidney vasculogenesis.

## 2 cc-RCC associated vasculogenesis

Solid tumors are characterized as multiscale, open, complex, and dynamic systems. They are complex due to the presence of numerous interacting components; dynamic because both the components and their interactions can undergo changes over time; and open because the tumor can freely communicate with surrounding and distant host tissue. Significant intratumor variations frequently occur (86). Tumor blood vessel development and vascular network diversity play a crucial role in tumor progression and spread into secondary metastatic foci (85). Angiogenesis recruits most clear-cell renal cell carcinoma (cc-RCC) blood vessels from peritumor and/or intratumor preexisting vessels (87). Previous studies have described additional mechanisms of cc-RCC vascularization as vasculogenic mimicry (malignant cells ability to delineate pseudo-vascular structures favouring metastasis) (88, 89), and tumor vasculogenesis has also been reported in other malignancies (90–92).

### 2.1 Sources and phenotype heterogeneity of EPCs contributors to cc-RCC vasculogenesis

**Figure 2:**
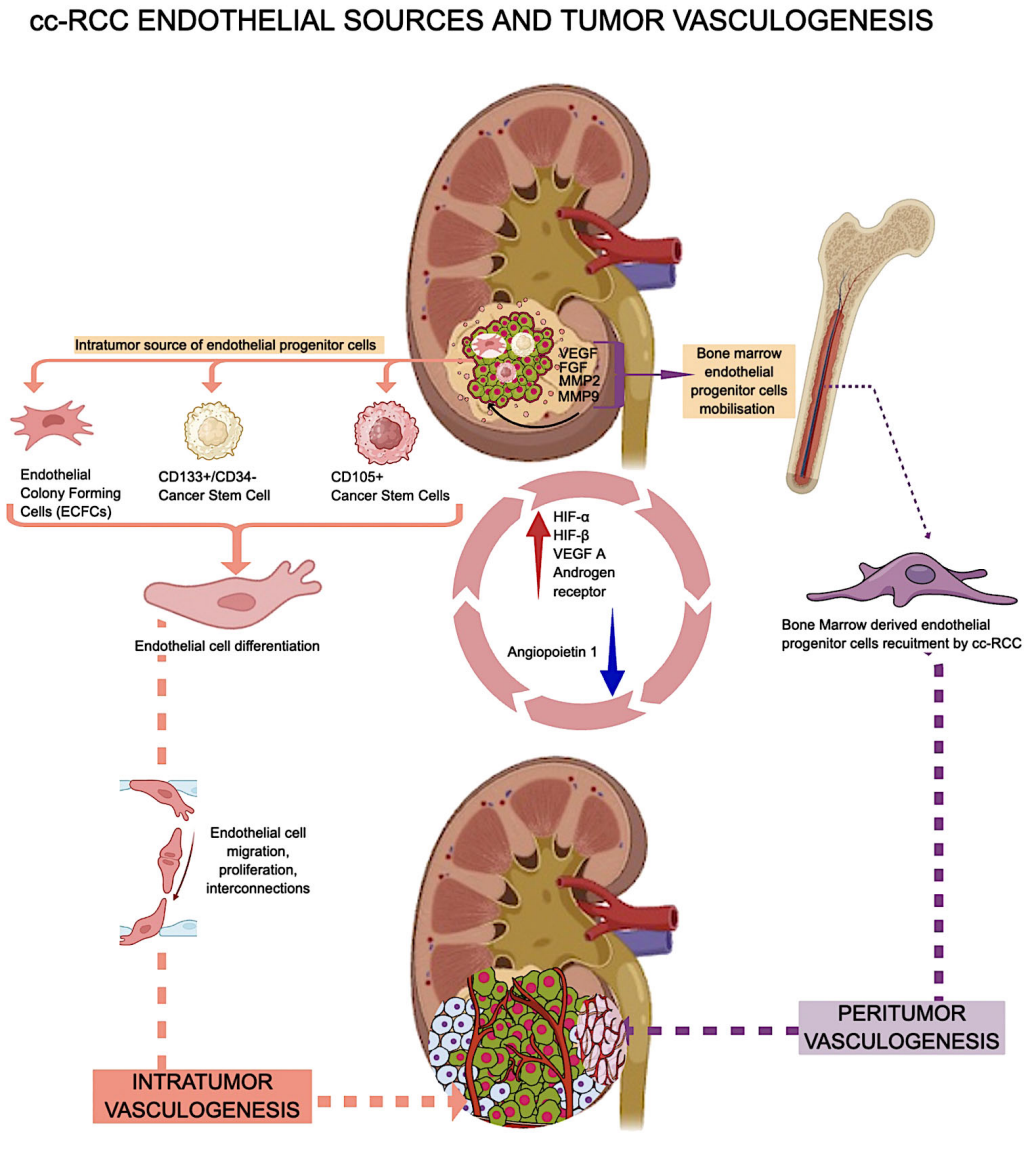
cc RCC vasculogenesis related to endothelial cells sources and distribution.

Researchers reported cc-RCC vasculogenesis in murine experimental models with von Hippel Lindau mutations (92). By using immunofluorescence to show where human-specific HLA markers and non-specific CD31 were located in the xenografts, Zhuang et al. provided more proof that tumor-derived endothelial cells are present inside the tumor. Based on this finding, the presence of an intratumor hemangioblast is most likely the cell that creates new blood vessels inside the tumor (92). In addition to this intrarenal potential source of endothelial cells with vasculogenic potential, cc-RCC uses extrarenal sources of EPCs to build its own vascular network. Cancer cells release several pro-angiogenic cytokines into the bloodstream, attracting several subtypes of bone marrow-derived endothelial progenitor cells (EPCs). During the initial stages of tumor development, EPCs sustain the angiogenic switch, a phenomenon known as vasculogenesis (93). Some authors suggested that cc-RCC peritumor and intratumor vasculogenesis have different EPC sources. The research by Poletto et al. (94), says that EPCs from bone marrow start and maintain peritumor vasculogenesis and angiogenesis, while ECFCs in the cc-RCC start and maintain intratumor vasculogenesis. RCC-derived ECFCs can multiply and make new tubes just like other EPCs, but they are less likely to die when rapamycin is added (95). Previous publications have shown that normal and cancer stem cells are involved in cc-RCC-related vasculogenesis. Bussolatti et al. isolated a group of CD133+/CD34-cells with a high ability to differentiate into endothelial cells *in vitro* and to contribute to *de novo* tumor vessel development *in vivo* (96). The same group also looked at CD105-positive cc-RCC cancer stem cells and discovered that they can change into endothelial cells and have the potential to become blood vessels (97). For example, the CD105+ group of cancer stem cells can release exosomes and microvesicles that contain certain messenger RNAs (mRNAs), like VEGF, FGF, MMP2, and MMP9. These molecules are critical in facilitating processes such as intratumor vasculogenesis and metastatic niche formation. Additionally, they contribute to the inhibition of T cell activation and dendritic cell activation, thereby affecting immune responses (98–100). Some situations, like low oxygen and being stimulated by angiogenic growth factors, make it easier for CD105-positive endothelial precursors to release exosomes that can help blood vessels grow (101). RCC-derived cancer stem cells can self-renew and contribute to tumor vasculogenesis (102). It is completely unknown what role kidney-derived mesenchymal stem cells play in cc-RCC vasculogenesis (103). **Figure 2** presents a summarized overview of cc-RCC vasculogenesis endothelial sources and vasculogenesis types.

### 2.2 Factors influencing cc-RCC vasculogenesis

**Figure 3:**
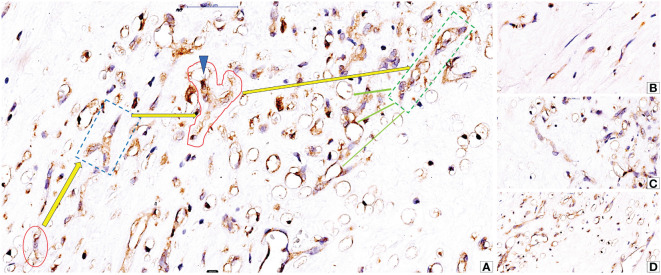
Microscopic evidence of CLIC1-positive isolated stromal cells with endothelial morphology (**A**, red oval) and their ability to organize into cords (**A**, interrupted blue line quadran and red area), to acquire philopodia (**A**, blue arrowhead) and to form hollows inside cytoplasm as prerequisite for lumen formation (**A**, green dotted line quadran and green arrows). Detailed aspects of isolated stromal CLIC1 positive cells **(B)**, CLIC1 positive cords **(C)** and lumenization **(D)** are presented in figures B to **(D)** Immunohistochemistry for CLIC1 performed by using anti-CLIC1 antibodies and diaminobenzidine as chromogen.

Several factors influence intratumor cc-RCC vasculogenesis. Hypoxia and HIF family proteins like HIF-α (1α, 2α, and 3α) and HIF-β (1β, 2², and 3β) help cancer cells grow by encouraging the growth of new blood vessels and angiogenesis (104). VEGF A is one of the most potent vasculogenic and angiogenic factors in both normal (105) and tumor conditions (106–109). High levels of VEGF A and low levels of Angiopoietin 1 (Ang1) cause a specific type of vasculogenesis that only happens in cc-RCC. This is how large blood vessels get into the tumor mass (108). In an experimental model of RCC with bone metastases, Xie et al. demonstrated that a similar vasculogenic process takes place in RCC bone metastases, and the decrease in Ang1 levels may be specific to RCC cell lines compared to other tumor cell lines (108). Androgen receptors in cc-RCC tumor cells positively correlated with intratumor-initiated vasculogenesis in cc-RCC patients (110). Researchers underestimate or completely neglect some vasculogenesis-associated factors in evaluating cc-RCC vasculogenesis. Chloride channels are one of them. CLIC-1 and CLIC-4 are the two main chloride channels that are highly expressed in endothelial cells and play a big role in the process of embryonic vessel development (111). Cutting down on CLIC1 stops not only the growth of endothelial cells in culture, but also their migration, the expression of integrins, and the formation of capillary networks (111). During postnatal life, CLIC1 expression in the kidney is present both in renal epithelial cells and stromal vascular endothelial cells. CLIC-1 is retained and overexpressed by cc-RCC tumor cells during malignant transformation, with its expression also observed on the stromal tumor blood vessel endothelium (112) (**Figure 3A**). Researchers detected isolated CLIC1-positive cells inside the tumor stroma as well as in between tumor cells (112). Researchers did not study the involvement of isolated CLIC1-positive stroma cells with endothelial morphology in the local initiation of tumor stroma vasculogenesis. CLIC1-positive stromal cells’ potential to serve as sources of peritumor vasculogenesis remains unstudied despite their high expression in tumor stroma cells. Recent microscopic evidence about the shape of stromal CLIC1-positive endothelial cells and how they are arranged into cords and tube-like structures seen in cc RCC strongly suggests that CLIC1 is involved in the formation of blood vessels around the tumor (**Figures 3B–D**).

## 3 Interrelation in between cc RCC vasculogenesis, tumor microenvironment and cc RCC adjacent normal tissue

**Figure 4:**
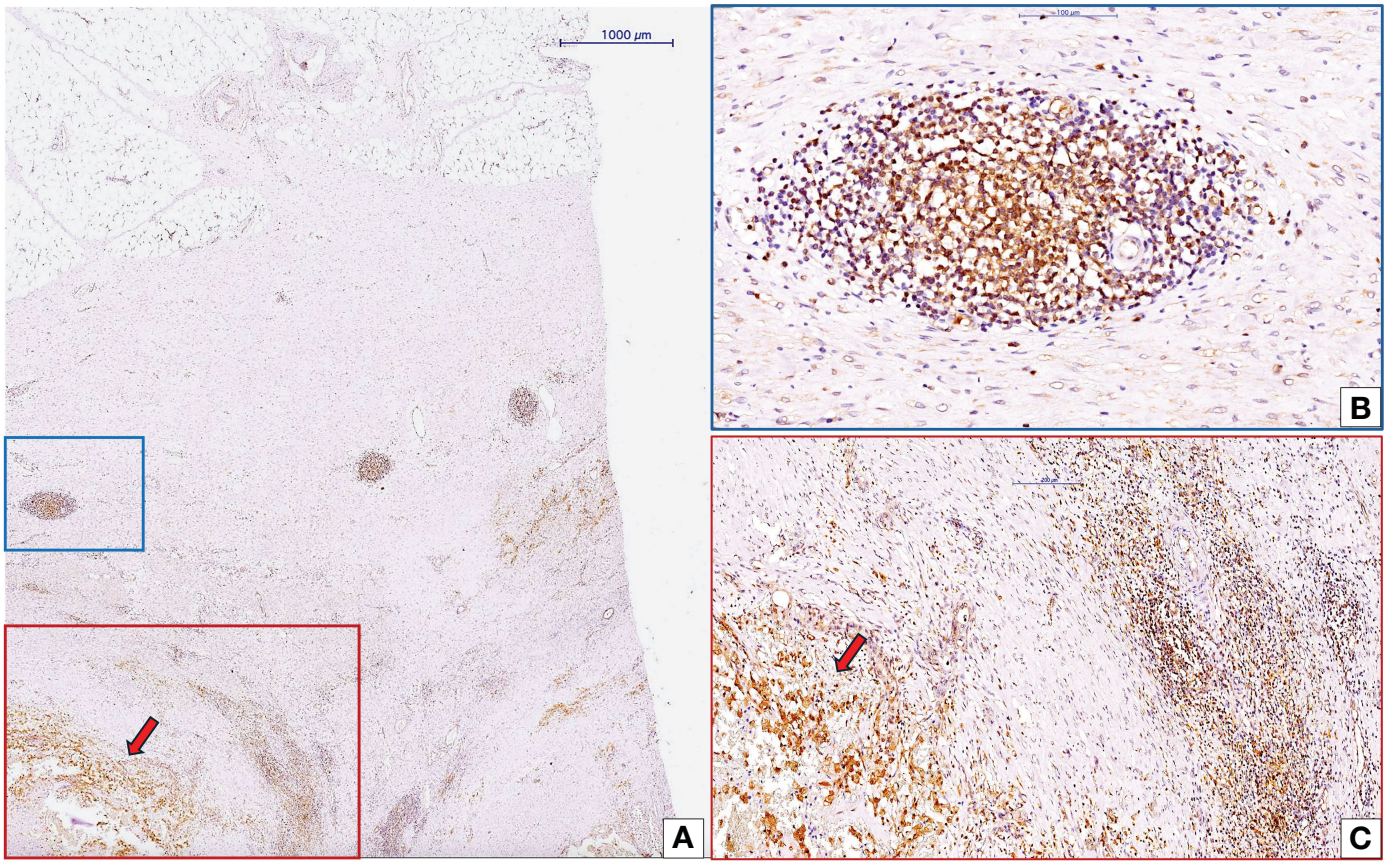
Interaction between CLIC1-positive cc-RCC and tumor microenvironment inflammatory components. Low magnification view of cc-RCC (red arrow) and adjacent stroma **(A)**. Inside the cc-RCC adjacent stroma we may identify to organizational pattern of inflammatory cells: tertiary lymphoid structure (TLS, blue quadran, **A**) and diffuse inflammatory infiltrate (red quadran, **A**). Detailed view of TLS **(B)** highlighted that around this structure the number of blood vessels is low sustaining previous data about the protective role of TLSs related to vasculogenesis/angiogenesis, tumor progression and metastasis. Diffuse inflammatory infiltrate close to tumor was detailed in panel **(C)** Immunohistochemistry for CLIC1 performed by using anti-CLIC1 antibodies and diaminobenzidine as chromogen.

cc-RCC blood vessel growth depends on endothelial progenitor cells coming from inside the tumor or the bone marrow (92–97). These cells must be present, differentiate, and/or be recruited. Most intratumor EPC sources are of stromal origin and may interact with other stromal components. There are three main parts of the tumor stroma: the vascular compartment, the cancer-associated fibroblast compartment, and the immune cell compartment. The extracellular matrix components are made in all three stromal compartments and include hyaluronan receptors, angiogenic/vasculogenic growth factors, and matrix metalloproteinases. Inflammatory cells within the immune cell compartment can diffusely infiltrate tumor stroma as tumor infiltrating lymphocytes (TILs) or organize into tumor-associated lymphoid structures at various developmental stages, known as tertiary lymphoid structures (TLS) (**Figures 4A–C**). A recent study validated TLSs as biomarkers with predictive roles for responsiveness to an immune checkpoint inhibitor-based drug regimen (113). The Society for Immunotherapy of Cancer Annual Meeting reported that treating patients with TLS-positive tumors with a PD-L1-targeted agent plus a multi-kinase inhibitor with anti-angiogenic activity led to clinical responses, even in types of cancer that are usually thought to be resistant to checkpoint inhibitors (113). Xu et al. (114) identified both tumor-proximal and tumor-distal TLS in cc-RCC. Tumor-proximal TLS localize between adjacent normal tissue and malignant areas of renal carcinoma (114). EPC numbers in RCC-adjacent tissues were significantly higher than those in control groups. EPCs gradually increased, along with tumor diameters (115). Tumor TLS inhibits EPC recruitment and may inhibit vasculogenesis. Studies have reported this in breast cancer (116), but not for cc-RCC. Researchers need to further investigate the interrelationship between TLSs and tumor vasculogenesis, a crucial aspect currently overlooked in tumor vasculogenesis research.

## 4 cc-RCC vasculogenesis impact on therapy response

Researchers are developing innovative current therapies as multitarget agents for several tumor components related to malignant cells and tumor stroma. The ability of malignant cells to change their phenotype during tumor progression, metastasis, or as a response to therapy is still an unresolved challenge in cancer therapeutic management. As we previously described, cc-RCC vasculogenesis is a highly heterogeneous process with a multifocal appearance (intratumor and peritumor areas) and different sources of EPCs.

### 4.1 CD105+ cancer stem cell-derived endothelial progenitor cells as a therapeutic target in cc-RCC

Brossa et al. (117) experimentally tested the effects of TRC 105 (antibodies against CD105) combined with or without Sunitib on the cc-RCC-derived tumor endothelial cells (TEC) and CD105+ cancer stem cell-derived endothelial progenitor cells (CSC-TEC). They showed that TRC105 hindered the capacity of TEC and CSC-TEC to arrange themselves into tubular structures, whereas it did not restrict their proliferation or survival. When TRC105 was co-administered with several anti-angiogenic medications, it had a synergistic effect only when combined with the tyrosine kinase inhibitor Sunitinib. TRC105 and Sunitinib together had similar effects on stopping the growth, survival, and tubulogenesis of both CSC-TEC and tumor-derived TEC. They demonstrated that the combination of TRC105 and Sunitinib caused the phosphorylation of Smad 2/3 at a molecular level, promoting endothelial cell death. Additionally, TRC105 increased Sunitinib’s ability to block VEGF signaling and decreased VEGFR2-Akt-Creb activation, showing that the two drugs work together in a way that is like how enzymes work. These results showed that blocking both the VEGF and TGF-β pathways at the same time might be a good way to treat renal cell cancer (117).

### 4.2 Tyrosine kinase inhibitors and cc-RCC vasculogenesis

Cancer stem cells contribute to the formation of blood vessels in tumors by directly differentiating into endothelial cells, which form new blood vessels through vasculogenesis. *In vivo*, anti-angiogenic strategies stopped mouse angiogenesis. *In vitro*, they also stopped the growth and survival of CSCs after they had changed into endothelial cells. VEGF-receptor inhibition with the non-specific tyrosine kinase inhibitor Sunitinib or the anti-VEGF-receptor 2 neutralizing antibody disrupted the process of endothelial differentiation *in vitro*, while VEGF blockade using Bevacizumab did not, indicating a VEGF-independent mechanism. Sunitinib reduced CSC-induced vasculogenesis *in vivo* through tyrosine kinase inhibition, whereas VEGF blocking with sFlk1 did not show the same effect. As a result, during endothelial differentiation under hypoxia, sunitinib but not bevacizumab blocked the hypoxia-inducible factor pathway induction. The current findings demonstrate how VEGF-receptor blockage and VEGF inhibition differ in their effects on tumor vasculogenesis. When a tumor lacks oxygen, blocking VEGFR stops the growth of new blood vessels in the tumor. However, blocking VEGF only seems to influence endothelial cells that have already differentiated (118).

### 4.3 Intracellular Ca2+ related pathways as potential additional targets in cc-RCC vasculogenesis

Endothelial colony-forming cells (ECFCs) are the only type of endothelial progenitor cells (EPCs) that can connect with new blood vessels that are growing in tumors and have endothelial properties. The intracellular calcium (Ca2+) system is very important for turning on endothelial colony-forming cells (ECFCs), and it changes shape in RCC-ECFCs that come from ECFCs. Specifically, RCC-ECFCs appear to experience a decrease in the concentration of calcium ions (Ca2+) in the endoplasmic reticulum (ER) (95). Store-operated Ca2+ entry (SOCE) regulates the formation, activation, and recruitment of human EPCs in cc-RCC through the interaction between the endoplasmic reticulum Ca2+ sensor, Stim1, and the plasmalemmal Ca2+ channels, Orai1 and TRPC1. EPCs derived from untreated RCC patients (RCC-EPCs) experience significant changes in their calcium signaling system. It significantly decreases the amount of calcium stored in the endoplasmic reticulum, reduces the expression of inositol-1,4,5-receptors (InsP3Rs), and increases the levels of Stim1, Orai1, and TRPC1. Another thing is that endothelial progenitor cells (EPCs) from tumor patients are much less sensitive to VEGF activation, as shown by lower gene expression and Ca2+ signaling. In contrast, SOCE’s pharmacological elimination inhibits cell growth and division. It seems that these results make us question whether VEGFR-2 is the right target for anti-angiogenic therapies. Instead, Orai1 and TRPC1 might be better choices (93). SOCE might be a good way to treat metastatic RCC, a type of cancer that doesn’t respond to common treatments like anti-VEGF inhibitors and anti-mammalian targets of rapamycin (mTOR) blockers, for reasons that are either genetic or acquired. The complete reorganization of the intracellular Ca2+ toolbox in T-ECFCs may be the main reason why standard treatments for people with RCC don’t work well enough or at all (119). In RCC-ECFC, SOCE pharmacological and genetic deletions suppress proliferation and tube formation. Several types of cancer, including cc-RCC, have been studied in phase I–III clinical trials that looked at how to block Orai1 with carboxyamidotriazole, a drug that targets both vascular endothelial cells and tumor-derived ECFC (120, 121).

## 5 Critical overview on interplay in between cc-RCC vasculogenesis and other angiogenic mechanisms

The key angiogenic mechanisms that have been extensively explored in malignant settings for various tumor types include intussusceptive microvascular expansion, vasculogenic mimicry, and vascular cooption. Researchers have not extensively documented the interaction between these mechanisms and cc-RCC vasculogenesis. Hess and colleagues conducted a comprehensive analysis of the shared pathways involved in both embryonic vasculogenesis and vasculogenic mimicry observed in melanoma, with a particular focus on Eph receptors and ligands (122). The researchers said that increasing Ephrin A 2 expression makes melanoma cells more aggressive and improves vasculogenic mimicry. In their study, Talaat et al. (123) emphasized that patients with RCC are prone to displaying a locally aggressive behavior and an unfavorable prognosis when their tumor tissues demonstrate elevated levels of EphA2 and Ki-67. In addition, these results support the idea that people with RCC may benefit from new treatment methods that focus on the EphA2 receptor to improve their prognosis. No previous reports have found any links between Eph A2 overexpression in cc-RCC cells and the vasculogenesis or vasculogenic mimicry in renal cancer. Additionally, there have been no reports on the relationship between Eph A2 expression and distinct types of cc-RCC endothelial progenitor cells. Recently, He and *colab.* reported that sunitinib therapy enhanced the transcription of a long non-coding RNA (lncRNA), specifically lncRNA-ECVSR, in order to increase the stability of estrogen receptor β (ERβ) mRNA. As a result, the heightened expression of ERβ can then act by increasing the transcription of HIF2-α. Significantly, the activation of lncRNA-ECVSR/ERβ/Hif2-α signaling by sunitinib led to an enhanced cancer stem cell (CSC) phenotype, which in turn promoted vasculogenic mimicry (VM). Sunitinib/lncRNA-ECVSR can also increase the expression of ERβ, which can change the expression of lncRNA-ECVSR through a positive feedback loop at the transcriptional level. Preclinical research utilizing RCC mouse xenografts showed that combining sunitinib with the small-molecule anti-estrogen PHTPP can enhance the effectiveness of sunitinib while reducing the production of VM. The findings of this study could aid in the development of new biomarkers and innovative treatments to more effectively track and inhibit RCC progression. Ability of cc-RCC CSCs to participate to vasculogenic mimicry (124) together with their ability to generate endothelial progenitor cells (97) need further studies for the elucidation of relationship in between cc-RCC vasculogenesis and vasculogenic mimicry. Androgen receptors overexpression in cc-RCC promotes vasculogenic mimicry and metastasis via modulating lncRNA-TANAR/TWIST1 signals but also tumor vasculogenesis (110). Vascular cooption (VCO) was found to have an impact on cc-RCC metastases response to sunitinib (125) but a direct link in between cc-RCC vasculogenesis and VCO has not been previously published. Intussusceptive angiogenesis seems to be an angio-adaptative mechanism of cc-RCC to vatalanib therapy in murine experimental model (126) but no data on human cc-RCC are available.

## 6 Concluding remarks

Renal tissue is highly heterogeneous in relation to its vasculature in both normal and malignant conditions. Renal vasculature: During embryonic vasculogenesis, there are different types and origins of endothelial cells that come from both inside and outside the kidneys. However, during adulthood, vasculogenesis seems to be based only on cells that live inside the kidneys and can differentiate into endothelial cells as a way to help the blood vessel heal after being damaged by toxins or inflammation. During malignant transformation, cc-RCC is early vascularized, making it one of the most vascularized cancers in humans. Most studies have focused on tumor angiogenesis, leading to the utilization of several antiangiogenic and anti-vascular therapies in clinical practice as neoadjuvant therapy for cc-RCC. However, resistance to these therapies emerged relatively quickly, indicating the existence of additional mechanisms of tumor vascularization. cc-RCC vasculogenesis appears to be crucial for the development of therapy resistance due to various intratumor and extra-tumor cell sources with a high capacity to differentiate into endothelial progenitor cells and initiate both intratumor and peritumor vasculogenesis. Several new studies support cc-RCC vasculogenesis as a new target for new therapies to treat cases of cc-RCC that don’t respond to standard antiangiogenic and anti-vascular treatments. Researchers have already initiated clinical trials for agents targeting different steps of cc- RCC vasculogenesis.

## Author contributions

AAC: Funding acquisition, Methodology, Writing – original draft, Writing – review & editing. MF: Writing – review & editing. AN: Writing – original draft, Writing – review & editing, Funding acquisition, Investigation, Software. EM: Writing – original draft, Writing – review & editing, Conceptualization, Methodology. AF: Writing – original draft, Writing – review & editing. OF: Writing – original draft, Writing – review & editing, Investigation, Methodology. EC: Writing – original draft, Writing – review & editing, Formal analysis. SS: Writing – original draft, Writing – review & editing, Methodology. AMC: Conceptualization, Software, Supervision, Writing – original draft, Writing – review & editing.
